# Inhibition of hepatic p63 ameliorates steatohepatitis with fibrosis in mice

**DOI:** 10.1016/j.molmet.2024.101962

**Published:** 2024-05-28

**Authors:** Marcos F. Fondevila, Eva Novoa, Uxia Fernandez, Valentina Dorta, Tamara Parracho, Henriette Kreimeyer, Maria Garcia-Vence, Maria P. Chantada-Vazquez, Susana B. Bravo, Begoña Porteiro, Alba Cabaleiro, Mijra Koning, Ana Senra, Yara Souto, Joanne Verheij, Diana Guallar, Miguel Fidalgo, Abraham S. Meijnikman, Natalia da Silva Lima, Carlos Dieguez, Maria J. Gonzalez-Rellan, Ruben Nogueiras

**Affiliations:** 1 Department of Physiology, CIMUS, University of Santiago de Compostela, Santiago de Compostela, 15782, Spain; 2 CIBER Fisiopatologia de la Obesidad y Nutrición (CIBERobn), Santiago de Compostela, 15782, Spain; 3 Department of Medicine, University of California San Diego, La Jolla, CA, 92093, USA; 4 Proteomic Unit, Health Research Institute of Santiago de Compostela (IDIS), Santiago de Compostela, 15705, Spain; 5 Department of Pathology, Amsterdam University Medical Centers, Location AMC, Amsterdam, the Netherlands; 6 Department of Biochemistry and Molecular Biology, CIMUS, University of Santiago de Compostela, Santiago de Compostela, 15782, Spain; 7 Department of Medicine, Lunenfeld-Tanenbaum Research Institute, Mount Sinai Hospital, University of Toronto, Toronto, ON, M5T 3H7, Canada; 8 Galicia Agency of Innovation (GAIN), Xunta de Galicia, Santiago de Compostela, 15702, Spain

**Keywords:** p63, TAp63, MASLD, NAFLD, MASH, NASH, Metabolism, Steatosis, Fibrosis, Liver

## Abstract

**Objective:**

p63 is a transcription factor involved in multiple biological functions. In the liver, the TAp63 isoform induces lipid accumulation in hepatocytes. However, the role of liver TAp63 in the progression of metabolic dysfunction-associated steatohepatitis (MASH) with fibrosis is unknown.

**Methods:**

We evaluated the hepatic p63 levels in different mouse models of steatohepatitis with fibrosis induced by diet. Next, we used virogenetic approaches to manipulate the expression of TAp63 in adult mice under diet-induced steatohepatitis with fibrosis and characterized the disease condition. Finally, we performed proteomics analysis in mice with overexpression and knockdown of hepatic TAp63.

**Results:**

Levels of TAp63, but not of ΔN isoform, are increased in the liver of mice with diet-induced steatohepatitis with fibrosis. Both preventive and interventional strategies for the knockdown of hepatic TAp63 significantly ameliorated diet-induced steatohepatitis with fibrosis in mice fed a methionine- and choline-deficient diet (MCDD) and choline deficient and high fat diet (CDHFD). The overexpression of hepatic TAp63 in mice aggravated the liver condition in mice fed a CDHFD. Proteomic analysis in the liver of these mice revealed alteration in multiple proteins and pathways, such as oxidative phosphorylation, antioxidant activity, peroxisome function and LDL clearance.

**Conclusions:**

These results indicate that liver TAp63 plays a critical role in the progression of diet-induced steatohepatitis with fibrosis, and its inhibition ameliorates the disease.

## Highlights

• p63 is progressively elevated in mice with liver-induced steatohepatitis with fibrosis.
• Genetic knockdown of the isoform TAp63 in the liver ameliorates liver steatosis, inflammation and fibrosis in mice models of steatohepatitis, whereas TAp63 overexpression accelerates these events.
• Analysis in mice with gain and loss of function of hepatic TAp63 reveals multiple changes in the liver proteome.

## Graphical abstract

**Figure:**
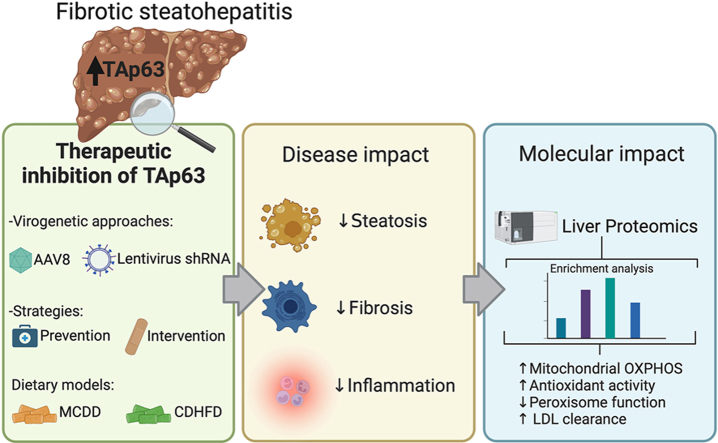

## 1 Introduction

Metabolic dysfunction-associated steatotic liver disease (MASLD) has become a major health challenge worldwide with a prevalence of 30% among adults [1]. MASLD encompasses a spectrum of liver disorders, starting from steatosis, which is primarily characterized by excessive fat accumulation in hepatocytes, with at least one of five cardiometabolic risk factors. It can advance to metabolic dysfunction-associated steatohepatitis (MASH), where inflammation and cellular injury, notably hepatocyte ballooning, are present, with or without fibrosis [2]. Liver fibrosis and steatohepatitis are the primary predictors of disease progression and patients with MASH and a fibrosis score ≥2 are at higher risk for liver-related events and mortality and are considered to have “at-risk” MASH [[3], [4], [5]]. Our understanding of the factors driving the progression of MASLD is evolving rapidly, yet we remain unable to definitively characterize which patients will develop advanced disease [6]. The significant clinical impact of MASLD, coupled with only one officially approved pharmacological option [7], highlights the urgent necessity to further understand its underlying mechanisms and the risks it poses to expand the offering and alternatives of efficient medications to tackle a disease as multifactorial and heterogeneous as MASLD.

The p53 family of transcription factors comprises p53, p63 and p73 [8]. They share structural and sequence similarities; however, each member can exert its own distinct functions via different mechanisms [[8], [9], [10]]. Functionally, beyond their widely known implication in cell cycle and cancer, this family plays a role in metabolism in health and disease [[11], [12], [13]]. In the liver, these transcription factors have been shown to play a causal role in metabolic disorders including MASLD [14,15].

There are two major isoforms of p63: TA variant (TAp63), which possesses an N-terminal transactivation domain; and ΔN variant (ΔNp63), with a truncated N-terminal transactivation domain [16,17]. The protein p63 controls differentiation, senescence and apoptosis in different cell types and organs [[18], [19], [20]]. In the liver, levels of TAp63 but not ΔNp63 are regulated by nutritional status in mice, decreasing upon fasting and increasing after consumption of a high-fat diet [14,21]. The liver-specific knockdown of p63 ameliorates the steatosis induced by four weeks of high fat diet, by reducing *de novo* lipogenesis, inflammation, and endoplasmic reticulum stress. By contrast, the liver-specific overexpression of TAp63 induces steatosis in mice fed a standard diet [14]. Further analysis in mice manipulating p63 in the liver revealed a novel role in the control of mitochondrial function. In particular, TAp63 increases the levels of ATG3, which in turn inhibits fatty acid oxidation and the oxidative phosphorylation in hepatocytes [22].

Regarding advanced MASH, which may be driven by very different mechanisms, the role of TAp63 remains less studied. A recent study in hepatic stellate cells (HSCs), which are the primary source of myofibroblasts that drive the fibrogenic process [23], showed that TAp63 increases in fibrogenic HSCs [24]. In addition, TAp63 protein levels in HSCs exhibit a positive correlation with both fibrosis stage and NAFLD Activity Score (NAS) in the liver of patients with MASH with fibrosis. Of note, mice lacking TAp63 in HSCs are protected against steatohepatitis with fibrosis in mice [24]. Other study showed that human liver TAp63 levels are progressively increased in MASLD, displaying positive correlation with the NAS [14]. However, p63 is expressed ubiquitously in all hepatic cells, and since the function of p63 in each of these cellular populations is unknown together with the fact that its silencing is to some extent dependent on the status of other family members as p53, it is difficult to predict what happens when manipulating this transcription factor in the whole liver in steatohepatitis with fibrosis.

In the present study, we show that hepatic TAp63 levels are increased in mouse models of steatohepatitis with fibrosis. The knockdown of TAp63 in the liver mitigates steatosis, inflammation, and fibrosis in mice with steatohepatitis with fibrosis induced by long-term feeding with methionine- and choline-deficient diet (MCDD) and choline deficient and high fat diet (CDHFD). In contrast, the overexpression of hepatic TAp63 accelerate the development of steatohepatitis with fibrosis. Unbiased proteomics analysis in the liver of these mice shows a landscape of multiple molecular pathways affected. These findings indicate that hepatic TAp63 plays a critical role in the development of steatohepatitis with fibrosis, and its inhibition in all liver cell types mitigates this disease.

## 2 Materials and methods

### 2.1 Animals and diets

8 weeks old male C57BL/6J mice were fed a standard diet (#U8200G10R, SAFE Diets), methionine-and choline-deficient diet (MCDD) (#A02082002BR, Research Diets), HFD (#D12451 45% fat, Research Diets), choline-deficient and high fat diet (CDHFD) (#D05010402; 45% fat, Research Diets) for the specified times. The detailed composition of each diet is included in Table S1. Food intake and body weight were monitored weekly during the experimental phase in all the experiments. Mice had *ad libitum* access to diets for the whole duration of the experiments until the moment of the sacrifice, in which we collected the blood, harvested the tissue samples, and weighted the organs. Animal protocols were approved by the Ethical Committee at the University of Santiago de Compostela.

### 2.2 Hepatic knockdown of p63 in mice

For the downregulation of p63 in the liver, we used two different mouse models. The first model consisted in the lentiviral TAp63 shRNA (1 × 10^9^ TU/ml) or control shRNA (1 × 10^9^ TU/ml) injection in wild type mice. In the second model, associated adenoviruses serotype 8 (AAV8) encoding GFP (AAV8-GFP) (SL100833, Signagen Laboratories) (1 × 10^10^ VG/ml) or Cre recombinase (AAV8-Cre) (SL100835, Signagen Laboratories) (1 × 10^10^ VG/ml) were injected into the tail vein of TAp63-floxed mice.

### 2.3 Lentivirus design and production

To downregulate p63 in the liver, specific shRNA sequences targeting TAp63 or schambled (control) were designed through the GPP Web Portal Tool (available at https://portals.broadinstitute.org/gpp/public/). The oligos targeting the transcripts of interest were synthesized and subcloned into pLKO.1 pure GFP vectors (Addgene) as previously described [25]. In brief, HEK293T cells were cultured in high-glucose DMEM supplemented with 10% FBS, 2 mM l-glutamine, and 1% penicillin and streptomycin. Cells were plated at a density of 8 × 10^6^ cells per 150 mm dish and transfected 24 h later with PEI (polyethylenimine; 408727, Sigma–Aldrich), along with 20 μg of pLKO.shRNAs plasmids, and 10 μg of psPAX2 and pMD2.G packaging mix. The medium was replaced after 24 h, and virus-containing supernatants were collected 48 h and 72 h post-transfection. Lentiviral particles were concentrated using centrifugal filter units with a 0.22 μm pore size (UFC903024, Amicon). The target sequences of the shRNAs used in this study were as follows:

shTAp63: 5′-TGTGAGTAACAATGACGTA-3′
shScrambled: 5′-CCTAAGGTTAAGTCGCCCTCG-3′

### 2.4 TAp63 overexpression *in vivo*

Overexpression of hepatic TAp63 was achieved by tail vein injection of adeno associated viruses serotype 8, AAV8-TAp63 (189SL100.865, SignaGen Laboratories) (1 × 10^10^ VG/ml), using AAV8-GFP (SL100.833, SignaGen Laboratories) (1 × 10^10^ VG/ml) for the control group [14].

### 2.5 Tail vein injection of virogenetic tools

To achieve a specific effect on the liver, viruses were injected into the tail vein. Mice were held in a specific restrainer for intravenous injections (Tailveiner TV-150, Bioseb). Injections into the vein were carried out using a 27 G × 3/8″ syringe. Mice were injected with 100 μL of adenoviral vectors diluted in saline.

### 2.6 Hepatocyte proliferation analysis

Mice were injected intraperitoneally with bromodeoxyuridine (BrdU) (100 mg/kg body weight) 3 h before the sacrifice to assess hepatocyte DNA synthesis [26]. Immunostaining for BrdU was then performed in liver sections and the number of BrdU-positive nuclei per field was determined.

### 2.7 Histological procedures

*Oil Red O staining*. Frozen liver samples were cut in 8 μm sections with a cryostat and stained in filtered Oil Red O for 10 min. After being washed in distilled water, sections were counterstained with Mayer's hematoxylin for 3 min and mounted in aqueous mounting (glycerin jelly).

*Sirius Red staining*. Samples fixated in paraffin were dewaxed, hydrated and stained in PicroSirius staining red for 1 h. Then, samples were washed with distilled water, dehydrated in three changes of 100% ethanol and cleared in xylene and mounted in a resinous medium.

*Immunohistochemistry.* For Collagen 1, Ki67 and BrdU staining, samples fixated in paraffin were dewaxed, hydrated, pre-treated in PTLink TE buffer pH 9 and blocked with 3% peroxidase for 10 min. Sections were then incubated with the primary antibody (ab260043, Abcam, 1:500) (dakoM7248, DAKO, 1:500) (555627, BD, 1:1000) overnight and at room temperature, followed by an incubation with the secondary antibody (EnVision, DAKO) for 30 min at room temperature. After that, DAB developer was used for 1 min and sections were counterstained with Mayer's hematoxylin for 10 min, dehydrated and mounted.

In these three histological staining techniques, up to 4 representative microphotographs of each liver section from every animal were taken with a BX51 microscope equipped with a DP70 digital camera (Olympus). Lipids in Oil Red O-stained sections, collagen depositions in Sirius Red-stained sections and positive areas in immunohistochemistry were quantified using Image J 2.9.0 software.

### 2.8 Assessment of NAFLD Activity Score (NAS)

Liver sections embedded in paraffin were stained with hematoxylin–eosin and Sirius Red. These sections were evaluated in tandem by experts trained in MASLD scoring using the NAFLD Activity Score (NAS) [27]. The NAS score was calculated as the unweighted sum of steatosis (0–3), lobular inflammation (0–3), and ballooning (0–2). Fibrosis was scored on a scale ranging from 0, 1a, 1b, 1c, 2, 3, to 4.

### 2.9 Hydroxyproline assay

Collagen content in the liver was evaluated by measuring the levels of hepatic hydroxyproline with the Hydroxyproline Assay Kit (MAK008, MERCK) following the manufacturer's instructions. Briefly, liver samples were hydrolyzed in concentrated hydrochloric acid at 120 °C for 3 h, and then evaporated to dryness under vacuum. We added chloramine T/oxidation buffer mixture and incubated it for 5 min at room temperature, then DMAB reagent for 90 min at 60 °C. Absorbance was determined at 560 nm and the amount of hydroxyproline was expressed as percentage of the control group.

### 2.10 Serum levels of metabolites

During the sacrifice of mice, whole trunk blood was collected and then spun for 15 min at 6000×*g* and 4 °C. The supernatant was transferred to a new tube to obtain the serum. Serum ALT activity (41283, Spinreact), AST activity (41273, Spinreact), cholesterol levels (1001093, Spinreact), triglycerides levels (1001310, Spinreact), free fatty acids levels (436-91995, 434-91795, WAKO), glucose levels (1001190, Spinreact) and insulin levels (EZRMI, MERCK/Millipore) were measured by spectrophotometry in a ThermoScientific Multiskan GO spectrophotometer.

### 2.11 Triglycerides content in the liver

Hepatic triglycerides were extracted as previously described [28]. In brief, 100 mg of tissue was homogenized in chloroform-methanol (2:1, vol/vol) for 3 min at 4 °C, then extracted by shaking for 3 h at room temperature. For phase separation, distilled water was added, samples were centrifuged, and the organic phase was obtained. The organic solvent remained to dryness under the vacuum at room temperature. The content of triglycerides in each sample was then measured in duplicate by spectrophotometry (1001310, Spinreact) and corrected by the sample mass, expressing the data as mg triglycerides per g of liver tissue.

### 2.12 Real time PCR

RNA was extracted using Trizol reagent (15596018, Invitrogen). 100 ng of total RNA was used for each RT reaction, and cDNA synthesis was performed using the M-MLV Reverse Transcriptase (28025013, ThermoFisher) and random primers. For real time PCR, a QuantStudio 5 Real-Time PCR Instrument (Applied Biosystems) and SYBR green Precision®PLUS reagent (PPLUS-LR, PrimerDesign) were used. The PCR cycling conditions included an initial denaturation at 95 °C for 3 min followed by 40 cycles at 95 °C for 5 s and 60 °C for 32 s. The oligonucleotide specific primers are shown in Table S2. All reactions were performed in duplicate. Expression levels were normalized to *Hprt* for each sample and expressed in relation (%) to the control group.

### 2.13 Quantitative proteomic analysis

*Protein identification by LC–MS/MS.* After liver protein extraction from the different mouse models, an equal amount of protein (100 μg) from all samples was loaded on a 10% SDS-PAGE gel. The run was stopped as soon as the front had penetrated 3 mm into the resolving gel. The protein band was detected by Sypro-Ruby fluorescent staining (Lonza), excised, and processed for in-gel, manual tryptic digestion as described elsewhere [29,30]. Peptides were extracted by carrying out three 20-min incubations in 40 μL of 60% acetonitrile dissolved in 0.5% HCOOH. The resulting peptide extracts were pooled, concentrated in a SpeedVac, and stored at −20 °C.

*Protein quantification by SWATH (Sequential Window Acquisition of all Theoretical Mass Spectra).* The MS/MS spectral libraries were created using data-dependent acquisition (DDA) approach by micro-LC–MS/MS as previously described our group [29,30]. To get a good representation of the peptides and proteins present in all samples, pooled vials of samples from each group were prepared using equal mixtures of the original samples. 4 μL (4 μg) of each pool was separated into a micro-LC system Ekspert nLC425 (Eksigen, Dublin, CA, USA) using a column Chrom XP C18 150 × 0.30 mm, 3 mm particle size and 120 Å pore size (Eksigent, SCIEX), at a flow rate of 5 μL/min. Water and ACN, both containing 0.1% formic acid, were used as solvents A and B, respectively. The gradient run consisted of 5%–95% B for 30 min, 5 min at 90% B and finally 5 min at 5% B for column equilibration, for a total run time of 40 min. When the peptides eluted, they were directly injected into a hybrid quadrupole-TOF mass spectrometer Triple TOF 6600 (Sciex, Redwood City, CA, USA) operated with a data-dependent acquisition system in positive ion mode. A Micro source (Sciex) was used for the interface between microLC and MS, with an application of 2600 V voltage. The acquisition mode consisted of a 250 ms survey MS scan from 400 to 1250 m/z followed by an MS/MS scan from 100 to 1500 m/z (25 ms acquisition time) of the top 65 precursor ions from the survey scan, for a total cycle time of 2.8 s. The fragmented precursors were then added to a dynamic exclusion list for 15 s; any singly charged ions were excluded from the MS/MS analysis. After MS/MS analysis, data files were processed using ProteinPilot™ 5.0.1 software from Sciex which uses the algorithm ParagonTM for database search and Progroup™ for data grouping. Data were searched using a Mouse specific Uniprot database, specifying iodoacetamide as Cys alkylation. The false discovery rate (FDR) was set to 1 for both peptides and proteins [31].

*Relative quantification by SWATH acquisition.* SWATH–MS acquisition was performed on a TripleTOF® 6600 LC–MS/MS system (AB SCIEX). Samples were analyzed using a data-independent acquisition (DIA) method. Each sample was analyzed using the LC–MS equipment and LC gradient described above for building the spectral library but instead using the SWATH-MS acquisition method. The method consisted of repeating a cycle that consisted of the acquisition of 100 TOF MS/MS scans (400–1500 m/z, high sensitivity mode, 50 ms acquisition time) of overlapping sequential precursor isolation windows of variable width (1 m/z overlap) covering the 400–1250 m/z mass range with a previous TOF MS scan (400–1500 m/z, 50 ms acquisition time) for each cycle. Total cycle time was 6.3 s. For each sample set, the width of the 100 variable windows was optimized according to the ion density found in the DDA runs using a SWATH variable window calculator worksheet from Sciex.

*Proteomics data analysis.* The targeted data extraction of the fragment ion chromatogram traces from the SWATH runs was performed by PeakView (version 2.2) using the SWATH Acquisition MicroApp(version 2.0). Up to ten peptides per protein and seven fragments per peptide were selected, based on signal intensity; any shared and modified peptides were excluded from the processing. Five-minute windows and 30 ppm widths were used to extract the ion chromatograms; SWATH quantitation was attempted for all proteins in the ion library that were identified by ProteinPilot with an FDR below 1%. The integrated peak areas (processed. mrkvw files from PeakView) were directly exported to the MarkerView software (AB SCIEX) for relative quantitative analysis. Marker view after normalization performed a Student's t-test analysis for comparison among the samples based on the averaged area sums of all the transitions derived for each protein. Proteomics data was analyzed with R using the R-packages “protti” [32] and “clusterProfiler” [33,34]. After normalizing the data, difference in abundance were calculated for each dataset respectively. Further we performed overenrichment analysis using GOEnrichment terms and performed Reactome Pathway Analysis for up-and downregulated proteins. For the Reactome Pathway analysis of proteomics data, a multiple testing correction using the Benjamin-Hochberg method was performed [35]. Upregulated proteins were defined as diff abundance >0.6 and downregulated <−0.6. The mass spectrometry proteomics data have been deposited to the ProteomeXchange Consortium via the PRIDE partner repository with the dataset identifier PXD050023.

### 2.14 Western blot

Total protein lysates from liver (20 μg) were subjected to SDS-PAGE, electrotransferred onto polyvinylidene difluoride membranes and probed with the antibodies indicated in Table S3. For protein detection, horseradish peroxidase-conjugated secondary antibodies and chemiluminescence (Amersham Biosciences) were used. Membranes were exposed to radiograph film (Super RX Fuji Medical XRay Film, Fujifilm) and developed with developer and fixing liquids (AGFA) under appropriate dark room conditions. Protein expression was quantified by densitometric analysis with Image J 2.9.0p software. Protein levels were normalized to GAPDH for each sample and expressed in relation to the control group. Uncropped blots accompanied by the location of molecular weight markers are shown in Figure S2.

### 2.15 Data analysis and statistics

Data are expressed as mean ± standard error mean (SEM). Statistical significances were determined by two-tail Student's t-test when two groups were compared. For multiple comparisons, a one-way analysis of variance (ANOVA) followed by Bonferroni post hoc multiple comparison test was performed. P < 0.05 was considered significant for all the analyses. Analyses were performed with the Prism Software Version 10.1.0 (GraphPad).

## 3 Results

### 3.1 Hepatic p63 is progressively increased in mice with diet-induced steatohepatitis with fibrosis

**Figure 1:**
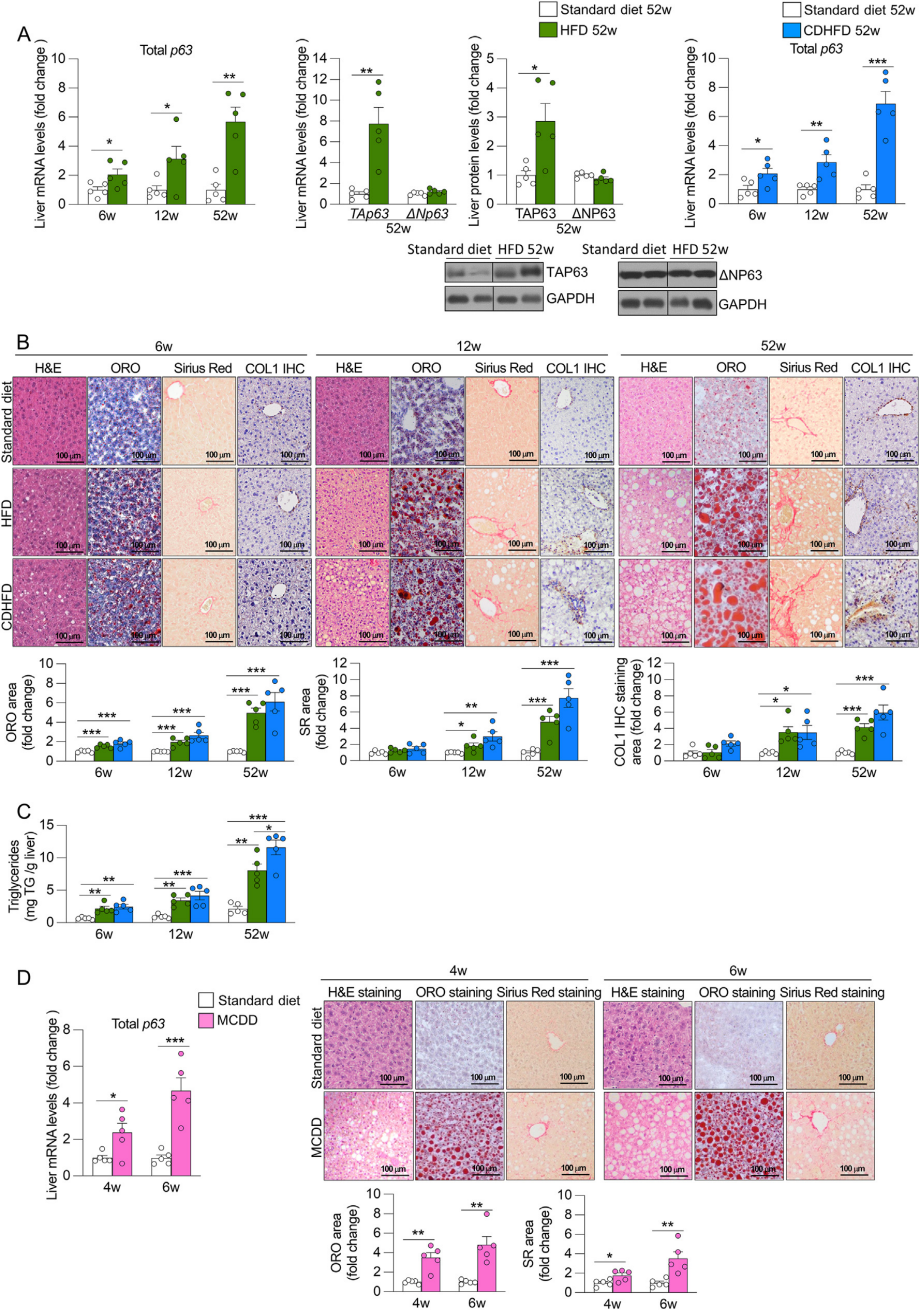
**Hepatic p63 is progressively increased in different mouse models of steatohepatitis with fibrosis**. (A) Expression of total p63, TAP63 and ΔNP63, (B) H&E staining, ORO staining, Sirius Red staining and Collagen 1 immunohistochemistry staining (COL1 IHC) in the liver of mice fed a HFD, CDHFD or standard diet for 6, 12, and 52 weeks (n = 5). (C) Hepatic triglycerides content in the liver of mice fed a HFD, CDHFD or standard diet for 6, 12, and 52 weeks (n = 5). (D) Expression of total p63, H&E staining, ORO staining and Sirius Red staining in the liver of mice fed a MCDD or standard diet for 4 and 6 weeks (n = 5). Staining areas were quantified. *Hprt* and GAPDH were used to normalize mRNA and protein levels. Data are mean ± SEM. ∗*p* < 0.05, ∗∗*p* < 0.01, ∗∗∗*p* < 0.001.

Liver p63 is upregulated in individuals with MASLD, displaying positive correlation with the NAS [14]. In mice, p63 levels are increased in diet induced-steatosis [14]. To study hepatic p63 levels in mice at advanced stages of the disease, we employed widely used dietary models, including a high fat diet (HFD), choline deficient and high-fat diet (CDHFD) and methionine- and choline-deficient diet (MCDD) for different periods [36]. We found that liver total p63 mRNA expression was increased after 6, 12 and 52 weeks of HFD and CDHFD, in comparison with the standard diet-fed control group (Figure 1A). The highest expression of p63 was detected at 52 weeks of HFD and CDHFD. Interestingly, this increase directly reflected a selective upregulation of its TAp63 isoform, as ΔNp63 levels remained unchanged (Figure 1A) under these conditions of liver damage characterized by advanced steatosis and fibrosis as shown in the specific liver staining for lipids (Oil Red O, ORO) and collagen (Sirius Red and Collagen 1 immunohistochemistry) (Figure 1B), and the increased hepatic triglycerides content (Figure 1C), in a context of obesity and associated metabolic dysfunction (Figure S1A) (Table S4). In the same line, mice fed a MCDD showed increased p63 mRNA expression after 4 and 6 weeks in parallel to the increased steatosis and fibrosis, with the highest expression at 6 weeks (Figure 1D). Our results indicate that total p63 levels, and specifically its TA isoform, are elevated in the liver of mice subjected to diet-induced steatohepatitis with fibrosis.

### 3.2 The hepatic knockdown of TAp63 prevents the development of steatohepatitis with fibrosis

**Figure 2:**
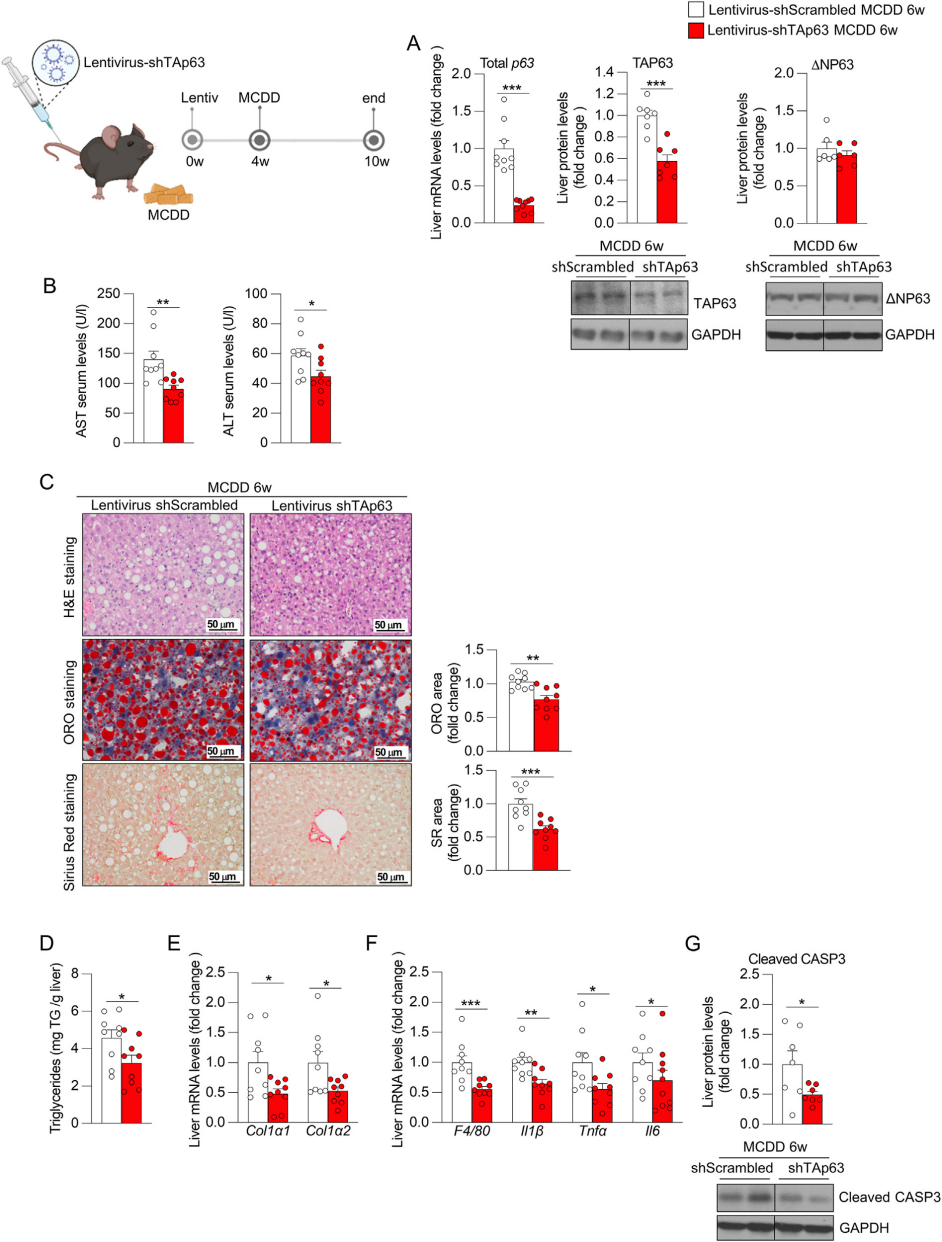
**Silencing of hepatic TAp63 in mice fed a a methionine- and choline-deficient diet (MCDD) for 6 weeks prevents the development of steatohepatitis with fibrosis. Mice receiving the tail vein injection of lentivirus encoding shRNA TAp63 or scrambled were fed a MCDD for 6 weeks**. (A) Hepatic expression of total p63 and protein abundance of TA and ΔN isoforms of P63 (n = 6–9). (B) Serum levels of AST and ALT (n = 9). (C) Liver sections stained with H&E (top), ORO (medium) and Sirius red (bottom). Staining areas were quantified (n = 9). (D) Hepatic triglycerides content (n = 9). (E) Expression of fibrosis markers in the liver (n = 9). (F) Hepatic expression of inflammation markers (n = 9). (G) Protein levels of hepatic cleaved caspase 3 (n = 7). *Hprt* and GAPDH were used to normalize mRNA and protein levels. Data are mean ± SEM. ∗*p* < 0.05, ∗∗*p* < 0.01, ∗∗∗*p* < 0.001.

Given that the specific TA isoform of p63 is associated with the liver disease progression in human and mice, we hypothesized that inhibiting hepatic TAp63 could prevent the development of steatohepatitis with fibrosis. To test this, mice were injected in the tail vein with a lentivirus encoding a shRNA against TAp63 (shTAp63) to knockdown liver TAp63 expression or with a scrambled shRNA (shScrambled) as a control; four weeks post-injection, they were fed a MCDD for 6 weeks to induce steatohepatitis with fibrosis. As expected, shTAp63-treated mice had significant downregulated levels of hepatic total p63 and TAP63 without affecting ΔNp63 abundance (Figure 2A); of note, they also displayed reduced levels of serum AST and ALT (Figure 2B), indicative of reduced liver damage. At hepatic level, the inhibition of p63 reduced steatosis, as shown in the ORO staining and hepatic triglycerides content (Figure 2C–D). We also detected reduced hepatic fibrosis, as shown in the Sirius Red staining (Figure 2C) and reduced liver expression of Collagen alpha 1 (*Col1α1*) and Collagen alpha 2 (*Col1α2*) (Figure 2E). Furthermore, we detected reduced mRNA levels of different markers of inflammation (Figure 2F) and decreased protein levels of the apoptotic marker cleaved caspase 3 (CASP3) (Figure 2G), indicating attenuated inflammation and liver damage following the knockdown of TAp63.

**Figure 3:**
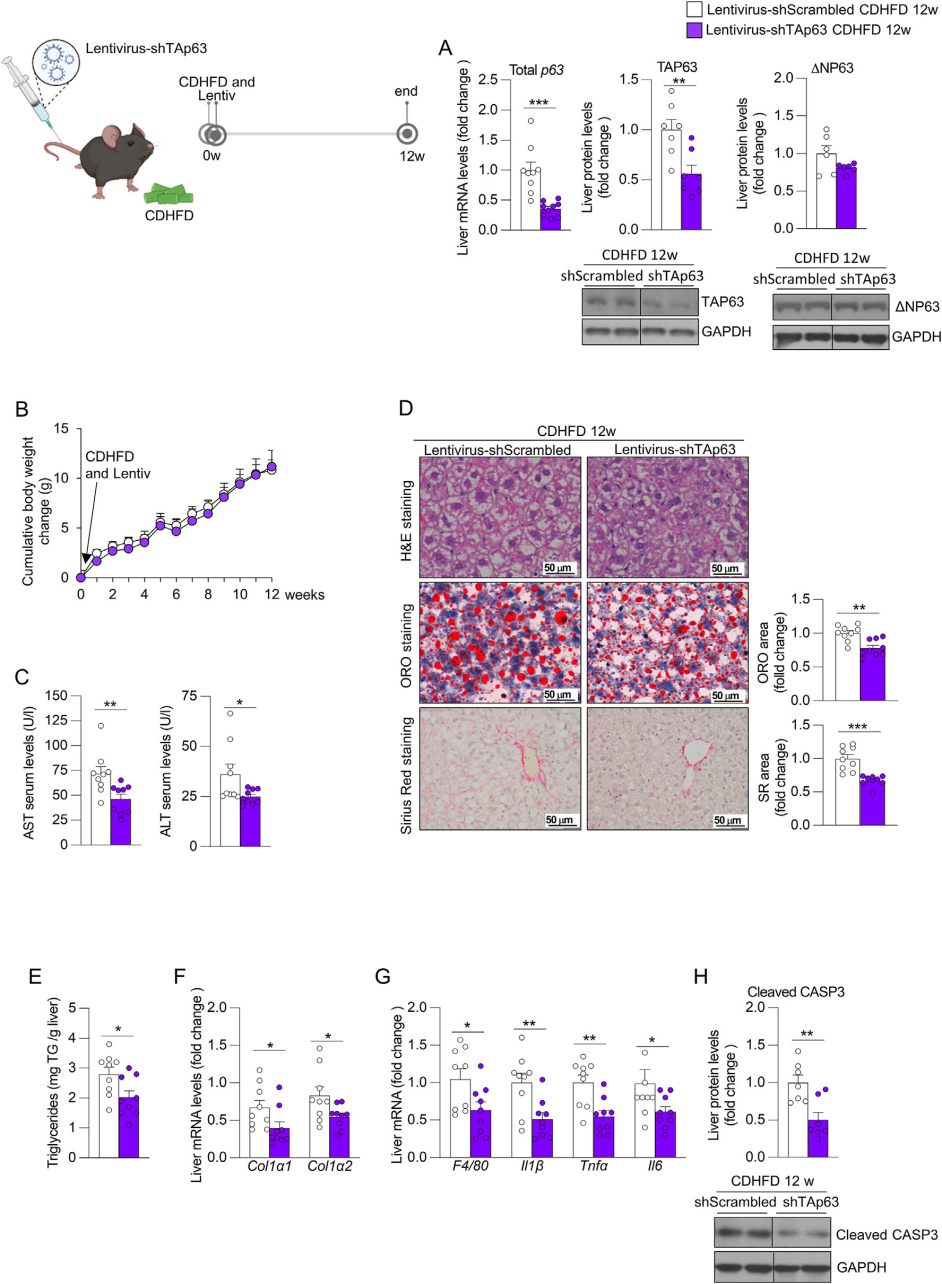
**Downregulation of hepatic TAp63 in mice fed a choline deficient and high-fat diet (CDHFD) for 12 weeks prevents the development of steatohepatitis with fibrosis**. Mice receiving the tail vein injection of lentivirus encoding shRNA TAp63 or scrambled were fed a CDHFD for 12 weeks. (A) Hepatic expression of total p63 and protein levels of TAP63 and ΔNP63 (n = 6–9). (B) Cumulative body weight change (n = 9). (C) Serum levels of AST and ALT (n = 9). (D) Liver sections stained with H&E (top), ORO (medium) and Sirius red (bottom). Staining areas were quantified (n = 9). (E) Hepatic triglycerides content (n = 9). (F) Expression of fibrosis markers in the liver (n = 9). (G) Hepatic expression of inflammation markers (n = 9). (H) Protein levels of hepatic cleaved caspase 3 (n = 7). *Hprt* and GAPDH were used to normalize mRNA and protein levels. Data are mean ± SEM. ∗*p* < 0.05, ∗∗*p* < 0.01, ∗∗∗*p* < 0.001.

We corroborated these results in a second model of steatohepatitis with fibrosis, in which mice receiving a tail vein injection of either shTAp63 or shScrambled were fed a CDHFD for 12 weeks. Indeed, mice with the hepatic knockdown of TAp63 exhibited reduced levels of hepatic total p63 and TAP63, serum AST and ALT, as well as decreased steatosis, fibrosis, inflammatory and apoptotic markers compared with the shScrambled group (Figure 3A–H). We did not detect changes in cumulative body weight gain, serum levels of triglycerides, cholesterol, glucose and epididymal adipose tissue mass, but we found reduced liver mass and increased serum levels of non-esterified fatty acids between the two groups (Figure 3B and Table S5).

### 3.3 The hepatic knockdown of TAp63 reverses the development of steatohepatitis with fibrosis

**Figure 4:**
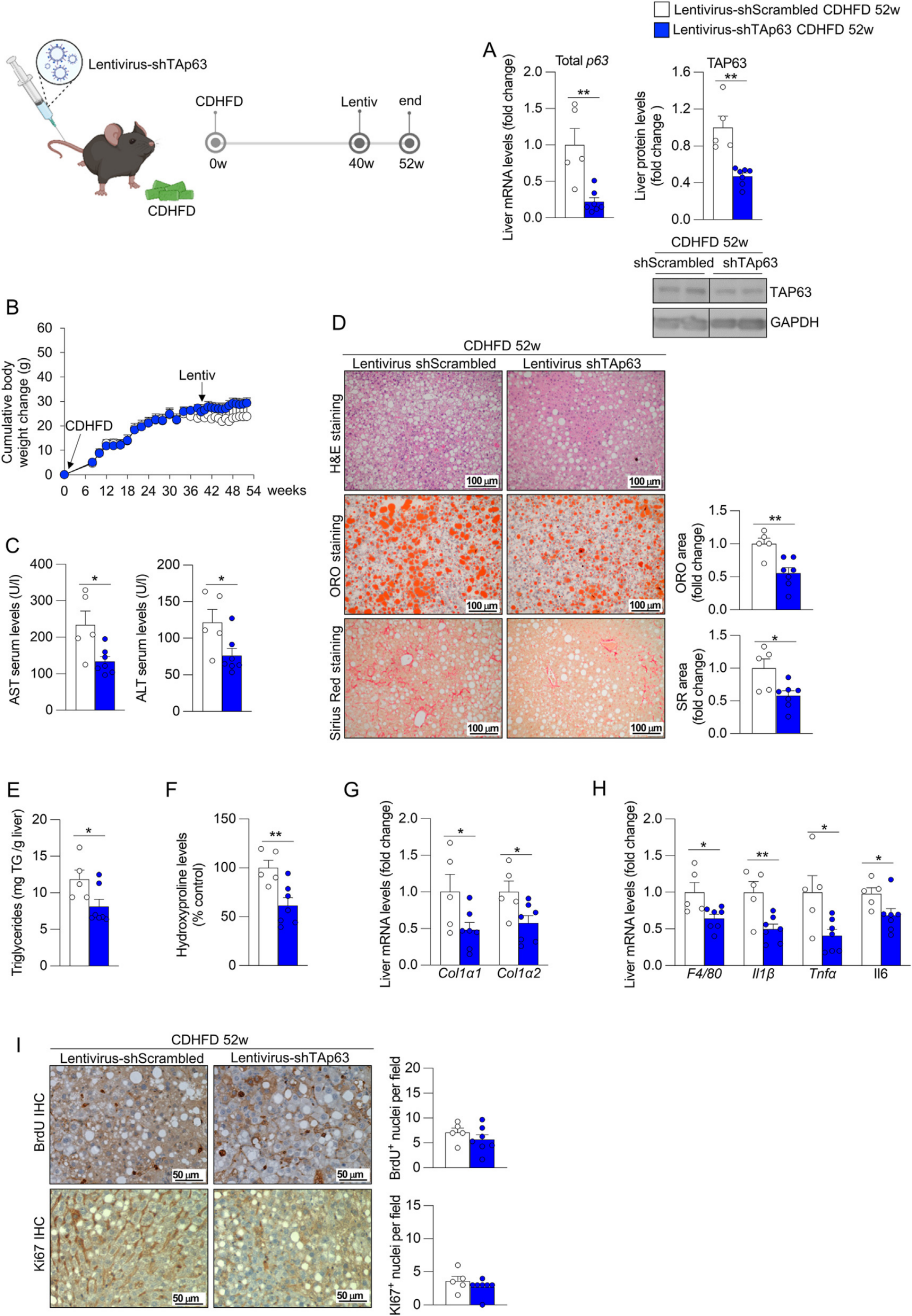
**Downregulation of hepatic TAp63 in mice fed a choline deficient and high-fat diet (CDHFD) for 52 weeks ameliorates the development of steatohepatitis with fibrosis**. Mice fed a CDHFD for 40 weeks received the tail vein injection of lentivirus encoding shRNA TAp63 (n = 5) or scrambled (n = 7) and were maintained in CDHFD for additional 12 weeks. (A) Hepatic expression of total p63 and protein levels of hepatic TAP63 and ΔNP63. (B) Cumulative body weight change. (C) Serum levels of AST and ALT. (D) Liver sections stained with H&E (top), ORO (medium) and Sirius red (bottom). Staining areas were quantified. (E) Hepatic triglycerides content. (F) Hepatic hydroxyproline levels. (G) Expression of fibrosis markers in the liver. (H) Hepatic expression of inflammation markers. (I) Immunohistochemistry staining liver sections of BrdU (top) and Ki67 (bottom). Number of staining-positive nuclei per field were determined. *Hprt* and GAPDH were used to normalize mRNA and protein levels. Data are mean ± SEM. ∗*p* < 0.05, ∗∗*p* < 0.01.

Next, we aimed to evaluate whether hepatic TAp63 inhibition could not only prevent but also reverse steatohepatitis with fibrosis. To study this, mice were fed a CDHFD for 52 weeks, receiving the tail vein injection of lentivirus encoding either shTAp63 or shScrambled at week 40. The downregulation of total p63 and TAP63 (Figure 4A) in the liver of shTAp63-treated mice did not affect body weight gain (Figure 4B), but led to reduced levels of serum AST and ALT (Figure 4C). In the liver, the inhibition of TAp63 reduced the lipid content, the triglycerides accumulation, and the total liver mass (Figure 4D–E and Table S6), and decreased fibrosis (Figure 4D), the hepatic hydroxyproline content (Figure 4F) and liver expression of Col1α1 and Col1α2 (Figure 4G). In addition, we found reduced expression of different inflammation markers (Figure 4H). Importantly, no changes were detected in hepatic cell proliferation, assessed by BrDU and Ki67 immunohistochemistry staining in the liver, nor in the serum levels of triglycerides, cholesterol, glucose and insulin, although we found an increase in non-esterified fatty acids in serum between the two groups (Figure 4I and Table S6).

**Figure 5:**
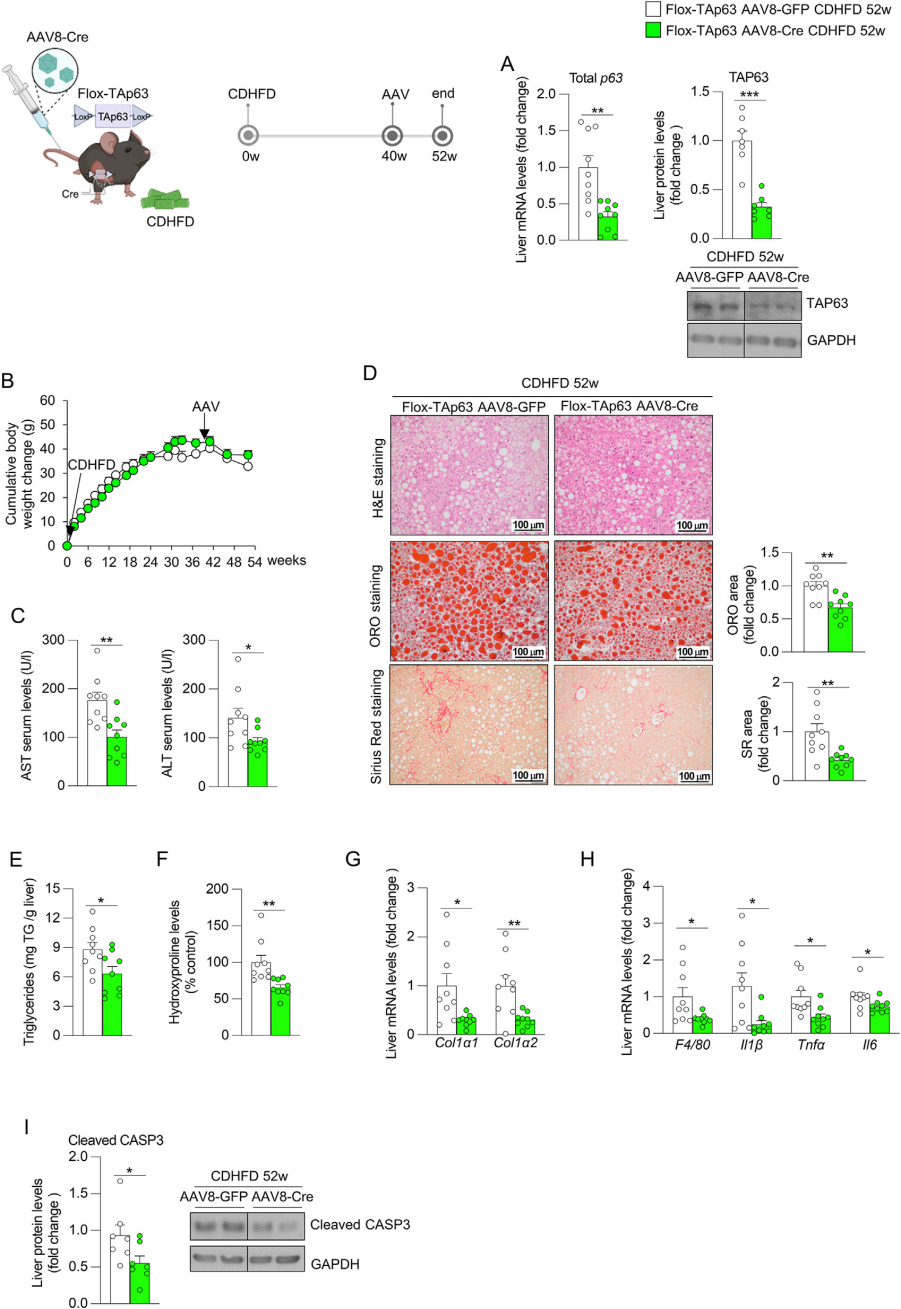
**Knockdown of hepatic TAp63 in mice fed a choline deficient and high-fat diet (CDHFD) for 52 weeks ameliorates the development of steatohepatitis with fibrosis**. TAp63-floxed mice were fed a CDHFD for 40 weeks, received the tail vein injection of adeno-associated virus serotype 8 (AAV8) encoding Cre recombinase or GFP, and were maintained in CDHFD for additional 12 weeks. (A) Hepatic expression of total p63 and protein levels of TAP63 (n = 7–9). (B) Cumulative body weight change (n = 9). (C) Serum levels of AST and ALT (n = 9). (D) Liver sections stained with H&E (top), ORO (medium) and Sirius red (bottom). Staining areas were quantified (n = 9). (E) Hepatic triglycerides content (n = 9). (F) Hepatic hydroxyproline levels (n = 9). (G) Expression of fibrosis markers in the liver (n = 9). (H) Hepatic expression of inflammation markers (n = 9). (I) Protein levels of hepatic cleaved caspase 3 (n = 7). *Hprt* and GAPDH were used to normalize mRNA and protein levels. Data are mean ± SEM. ∗*p* < 0.05, ∗∗*p* < 0.01.

Next, we aimed to corroborate these results in a second model of steatohepatitis with fibrosis, in which TAp63 floxed mice were fed a CDHFD for 40 weeks and then were injected with an adeno-associated virus serotype 8 (AAV8) encoding the recombinase Cre into the tail vein for additional 12 weeks to silence TAp63 expression in the liver [20]. Mice treated with AAV8-Cre showed significant reduced hepatic total p63 and TAP63 (Figure 5A) without changes in body weight gain (Figure 5B) and serum levels of triglycerides, cholesterol and insulin compared to the control group (Table S7). Concomitantly to the knockdown of TAp63, we detected a significant reduction in serum levels of AST and ALT (Figure 5C), hepatic lipids and triglycerides content (Figure 5D–E), and fibrosis, as indicated by lower collagen deposition, hydroxyproline levels and expression of fibrosis markers (Figure 5D, F, G), as well as reduced mRNA levels of inflammatory markers and protein levels of cleaved CASP3 (Figure 5H–I). Consistent with these results, steatosis, inflammation and ballooning components of NAS and fibrosis stage were reduced in this group too (Table S8). Curiously, mice silencing liver TAp63 showed reduced serum glucose levels and epididymal adipose tissue mass, and increased non-esterified fatty acids in serum, compared to the control group (Table S7). Overall, these results indicated that inhibition of hepatic TAp63 consistently ameliorated pre-developed steatohepatitis with fibrosis.

**Figure 6:**
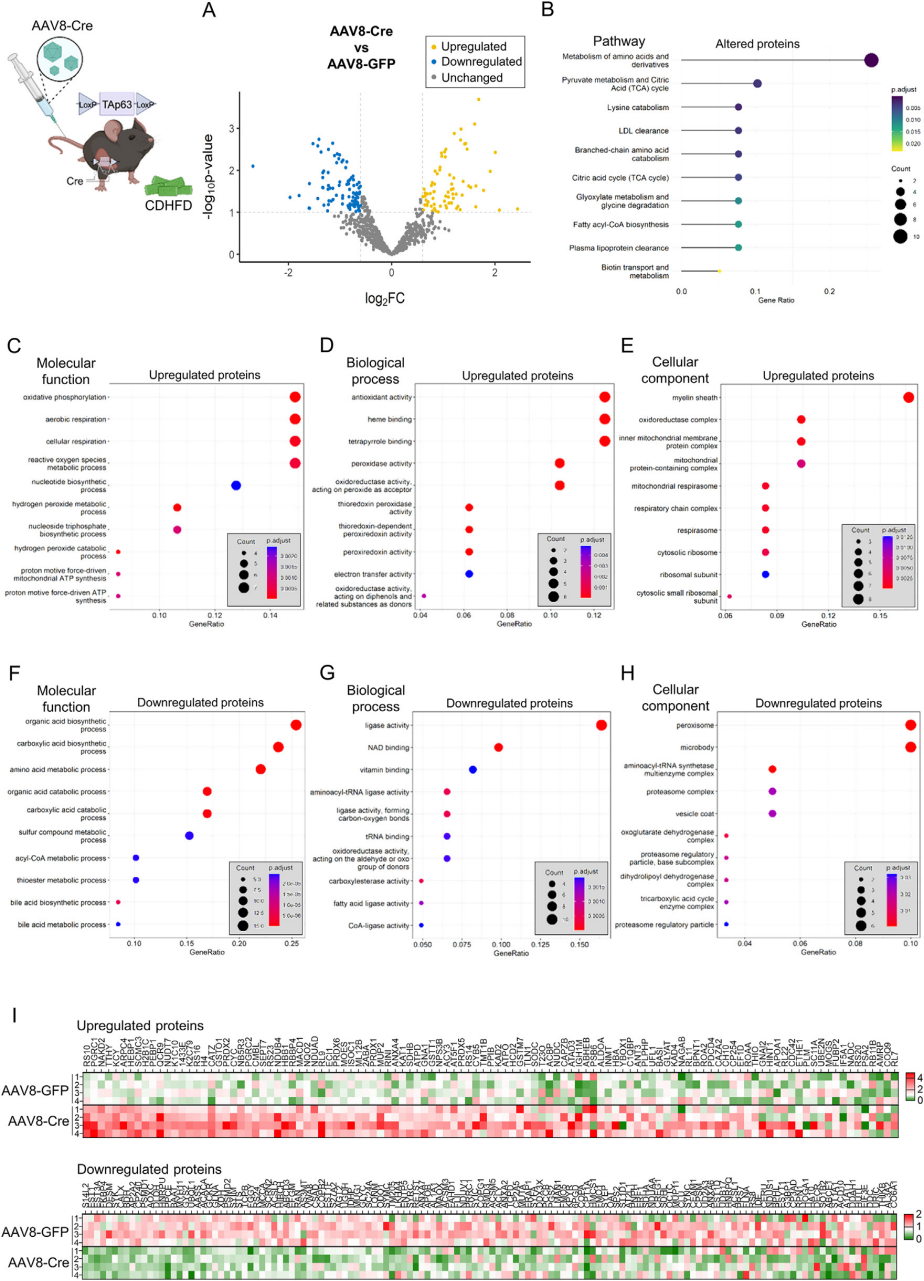
**Knockdown of hepatic TAp63 in mice fed a choline deficient and high-fat diet (CDHFD) for 52 weeks triggers changes in multiple proteins and pathways**. TAp63-floxed mice were fed a CDHFD for 40 weeks, received the tail vein injection of adeno-associated virus serotype 8 (AAV8) encoding Cre recombinase (n = 4) or GFP (n = 4), and were maintained in CDHFD for additional 12 weeks. (A) Volcano plots of hepatic protein expression determined by LC–MS/MS proteomics. (B) Reactome pathway analysis of proteins with altered abundance. Overrepresentation analysis of upregulated proteins according to the molecular function (C), biological process (D) and cellular component (E). Overrepresentation analysis of downregulated proteins according to the molecular function (F), biological process (G) and cellular component (H). (I) Heatmap of the upregulated and downregulated proteins.

To better characterize the changes triggered by the silencing of TAp63, we next performed proteomics studies in the liver of mice fed a CDHFD with knockdown of TAp63. Volcano plot showed multiples changes in the abundance of hepatic proteins between the two groups (Figure 6A); most of them were classified by Reactome as proteins related to multiple metabolic pathways, such as amino acids metabolism, citric acid cycle and LDL clearance. (Figure 6B). Then, we grouped the upregulated (111) and the downregulated (138) proteins in two sets and subjected them to GO-overrepresentation analysis. Among the upregulated proteins, oxidative phosphorylation, aerobic respiration, cellular respiration, and reactive oxygen species (ROS) metabolic process were identified as the top-ranked overrepresented molecular functions (Figure 6C). In the same line, proteins related to antioxidant activity in the biological process analysis, and to oxidoreductase complex and multiple mitochondrial components in the cellular component analysis, were upregulated in mice with downregulation of TAp63 (Figure 6D–E). On the other hand, among the downregulated proteins induced by TAp63 silencing, we found overrepresented proteins related to biosynthesis of organic acids and carboxylic acids function, ligase activity process and peroxisome cell component (Figure 6F–H). Heatmaps of the upregulated and downregulated proteins are provided in Figure 6I. Together, proteomics analysis revealed that the silencing of TAp63 increased the abundance of proteins involved in oxidative catabolism, which reinforces the known role of TAp63 inhibiting the mitochondrial function in hepatocytes [22], and in ROS mitigation, while decreasing those related to the biosynthesis of carboxylic acids and peroxisomes.

### 3.4 The overexpression of hepatic TAp63 in mice accelerates the development of advanced steatohepatitis with fibrosis

**Figure 7:**
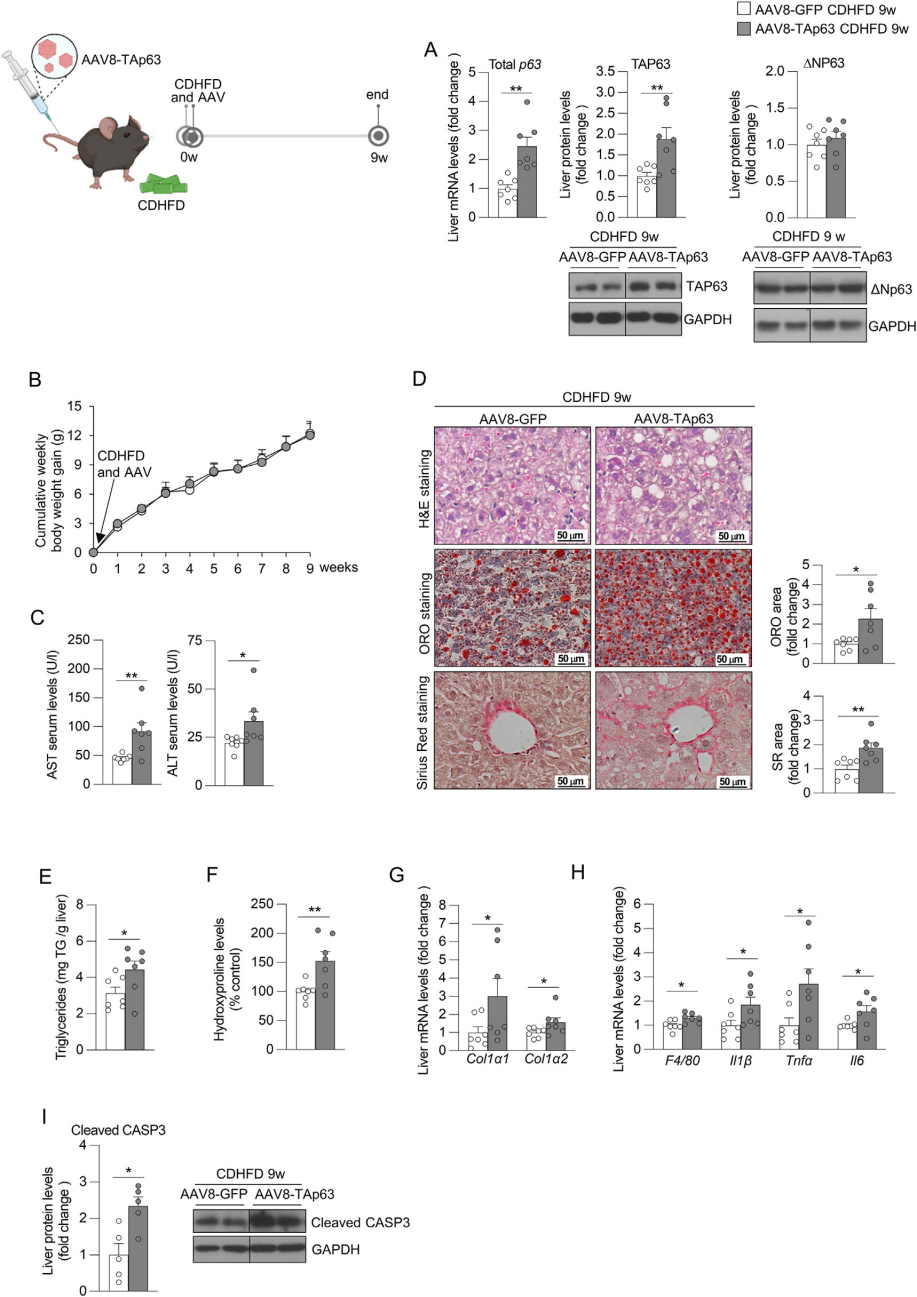
**Overexpression of hepatic TAp63 in mice fed a choline deficient and high-fat diet (CDHFD) for 9 weeks aggravates the development of steatohepatitis with fibrosis**. Mice receiving the tail vein injection of adeno-associated virus serotype 8 (AAV8) encoding TAp63 (n = 7) or scrambled (n = 7) were fed a CDHFD for 9 weeks. (A) Hepatic expression of total p63 and protein levels of TAP63 and ΔNP63. (B) Cumulative body weight change. (C) Serum levels of AST and ALT. (D) Liver sections stained with H&E (top), ORO (medium) and Sirius red (bottom). Staining areas were quantified. (E) Hepatic triglycerides content. (F) Hepatic hydroxyproline levels. (G) Expression of fibrosis markers in the liver. (H) Hepatic expression of inflammation markers. (I) Protein levels of hepatic cleaved caspase 3. *Hprt* and GAPDH were used to normalize mRNA and protein levels. Data are mean ± SEM. ∗*p* < 0.05, ∗∗*p* < 0.01.

After demonstrating that inhibition of TAp63 was sufficient to ameliorate advanced steatohepatitis with fibrosis, we next performed a gain-of-function experiment by overexpressing TAp63 in the liver of mice to investigate whether this would accelerate the development of the pathology. To achieve this, we injected AAV8 encoding TAp63 into the tail vein of mice and fed them a CDHFD for 9 weeks. We checked the efficiency of viral infection and found increased levels of total p63 mRNA and TAP63 protein, without altering ΔNp63 abundance (Figure 7A). Our results showed that the ectopic expression of TAp63 did not affect body weight gain (Figure 7B), but increased the serum levels of AST and ALT (Figure 7C). Overexpression of TAp63 also triggered the accumulation of lipids, triglycerides, collagen and hydroxyproline in the liver (Figure 7D–F), and increased the levels of hepatic markers of fibrosis, inflammation, and apoptosis (Figure 7G–I), without affecting the serum levels of triglycerides, cholesterol, non-esterified fatty acids, glucose, insulin, nor the epididymal adipose tissue mass (Table S9). In agreement with these results, steatosis, inflammation and ballooning components of NAS and fibrosis stage were increased in mice overexpression hepatic TAp63 (Table S10).

**Figure 8:**
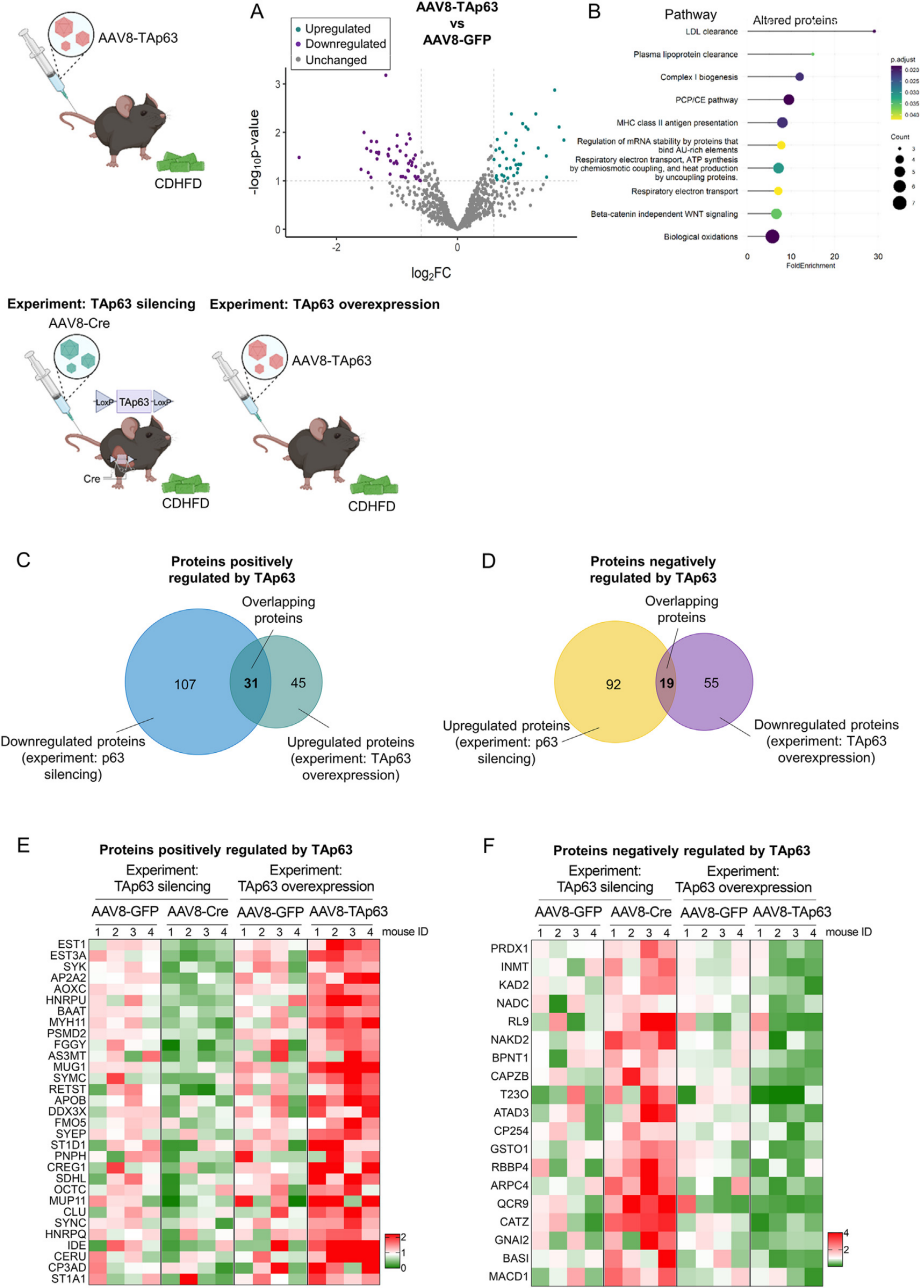
**Proteomics analysis in the liver of mice with overexpression of hepatic TAp63 or silencing of p63 revealed proteins consistently altered**. Mice receiving the tail vein injection of adeno-associated virus serotype 8 (AAV8) encoding TAp63 (n = 4) or scrambled (n = 4) were fed a CDHFD for 9 weeks. (A) Volcano plots of hepatic protein expression determined by LC–MS/MS proteomics. (B) Reactome pathway analysis of proteins with altered abundance. Venn's diagram of proteins positively (C) and negatively (D) regulated by TAp63 following the silencing of p63 and/or overexpression of TAp63. Heatmaps of positively (E) and negatively (F) regulated proteins in both experiments.

Further proteomics analysis revealed multiples changes in the abundance of hepatic proteins following the overexpression of TAp63 (Figure 8A), including proteins related to metabolism, specially to LDL clearance, plasma lipoprotein clearance and mitochondrial complex I (Figure 8B). Then, we specifically searched for proteins oppositely altered by TAp63 following its silencing and overexpression. For this, we combined proteomics data of both experiments silencing and overexpressing TAp63 in the liver. As shown in Venn's diagrams, we found that p63 positively regulated 31 proteins and negatively regulated 19 proteins (Figure 8C–D). The list of altered proteins and their abundance can be visualized in Heatmaps of Figure 8E–F and could constitute potential new mechanisms of action of p63 in the liver. Overall, these results indicated that hepatic TAp63 overexpression has an additive effect to CDHFD feeding in the progression of steatohepatitis with fibrosis to more severe stages of the disease.

## 4 Discussion

The present study expands our knowledge on p63 function in metabolic disease, indicating that liver p63 expression is progressively elevated in diet-induced steatohepatitis, showing consistency across three different mouse models. The knockdown of p63 in all cell types, and in particular de TA isoform in advanced stages of this chronic liver disease has a protective role. The importance of TAp63 in the development of steatohepatitis with fibrosis is in agreement with previous studies describing that i) hepatic TAp63 is already upregulated in mice with mild liver steatosis [14], ii) liver p63 in the liver and TAp63 in HSCs displays positive correlation with the NAS and other severity parameters in humans with MASLD [14,24], and iii) p63 expression remains high in hepatocellular carcinoma [37]. Together, this indicates that TAp63 could be involved in the progression of steatohepatitis with fibrosis throughout the whole spectrum of disease, a process governed by a complex network of mechanisms that remains to be completely understood [38]. Of note, the involvement of p63 in the progression of steatohepatitis with fibrosis seems to be specific to the TA isoform, as ΔNp63 lacks the TA domain and has opposite effects as TAp63 [[39], [40], [41]]. Consistent with previous studies showing that TAp63 but not ΔNp63 modulates lipid accumulation in hepatocytes [14] and fibrogenesis in stellate cells [24], now we found that the inhibition of TAp63 in the liver of mice exhibiting steatohepatitis with fibrosis exert beneficial effects in the disease progression. This is in line with the fact that the expression of TAp63 and ΔNp63 are controlled by distinct mechanisms in liver cells playing different biological roles since the former exhibit a truncated TA domain even behaving in some instances as a dominant negative [42].

Our studies manipulating TAp63 in the whole liver also suggest a global hepatic effect of TAp63 in steatohepatitis with fibrosis. While overexpression of TAp63 in the liver aggravates steatosis, inflammation, and fibrosis in steatohepatitis, the knockdown of hepatic TAp63 mitigates these effects. Importantly, metabolism, inflammation and fibrosis are closely intertwined and give account of underlying alterations in different hepatic cell types including hepatocytes, stellate cells, inflammatory cells and others [43]. Indeed, by using AAV8 and lentivirus, we are likely targeting various cell populations within the liver, so the observed phenotypes in mice with overexpression and knockdown of TAp63 indicate a combined result of altering this transcription factor in multiple hepatic cells. It is described that TAp63 in hepatocytes promotes triglycerides storage and inflammation [14], while TAp63 in HSCs stimulates the metabolic rewiring required for ATP production and fibrogenesis [24]. However, the potential role of TAp63 in other hepatic cell populations during the progression of steatohepatitis with fibrosis cannot be ruled out. However, the tools for targeting specific cell types are still scarce and from a translational perspective, it is highly challenging. The inhibition of TAp63 in different hepatic cells was precisely the aim of the study, as this might be of interest for future investigations trying to develop a pharmacological strategy targeting p63 in the liver irrespective of the cell population. Other relevant points related to the use of viral and virogenetic tools are the efficiency of targeting hepatic TAp63 and potential extrahepatic actions. We chose viral designs, doses and routes of administration based on previous experiments performed in our lab targeting TAp63 to find the optimal amount of virus efficiently silencing p63 in the liver without affecting its expression in other metabolically relevant organs of the mouse body [15,21]. Remarkably, the efficiency of TAp63 downregulation was lower than 80% across the different experiments of the project, depending on the model. This partial hepatic silencing was sufficient to trigger the beneficial effects on liver disease progression in multiple experiments, which could be a desirable characteristic for future investigations in pharmacological therapy.

Our findings highlight the potential benefit of targeting TAp63 in the liver to alleviate steatohepatitis with fibrosis. The hepatic inhibition of TAp63 in mice with steatohepatitis exerted beneficial reductions in steatosis, inflammation and fibrosis, which constitute together with ballooning the major features to assess the staging of MASLD by the gold-standard histological scoring system [27]. Remarkably, these beneficial effects were obtained independent of i) the selected dietary mouse model of disease and time of exposure (MCDD for 6 weeks, CDHFD for 12 weeks, CDHFD for 52 weeks), ii) the approach to target p63, achieving consistent results by using either lentivirus encoding shRNA against TAp63 and AAV8 encoding Cre recombinase in TAp63-floxed mice, and iii) the strategy utilized, attenuating the disease severity by using either prevention or intervention strategies. This consistency is especially important in the research of MASH, as the lack of robustness of results in preclinical models has been pointed out as key to explain why so many clinical trials in MASH recently failed [44].

Our results also provide potential mechanisms of action of TAp63 in steatohepatitis with fibrosis, as revealed by multiple changes in proteins and pathways induced by the manipulation of hepatic TAp63. Gain and loss of function of TAp63 consistently triggered changes in the levels of proteins mostly related to metabolism, in agreement with the known role of TAp63 in the control of metabolism in hepatocytes and HSCs [14,22,24]. Our data showed that, upon silencing of TAp63, upregulated proteins showed overrepresentation in oxidative phosphorylation, aerobic respiration and cellular respiration, further supported by the cellular component analysis showing overrepresentation of multiple mitochondrial categories. This is consistent with the already known role of p63 in the mitochondrial function in the hepatocytes, in which TAp63 controls the mitochondrial fatty acid oxidation via PGC1α, CPT1a and different OXPHOS complex subunits under steatosis, and suggests a tight control of mitochondrial function by TAp63 across the whole spectrum of steatohepatitis [22]. Interestingly, the silencing of TAp63 also upregulated proteins involved in antioxidant activity, a function which is largely unknown for p63 and completely unstudied for p63 in the liver [45]. Oxidative stress is a well-stablished contributor to the progression of MASH, and hence, multiple antioxidants are currently evaluated in clinical trials against MASLD [46]. In the same line, we detected downregulation of proteins related to peroxisomes in livers silencing TAp63. Peroxisomal oxidation of fatty acids generates oxidative stress and is known to contribute to the development of MASLD [47].

In addition, both the overexpression TAp63 and its downregulation led to alterations in levels of proteins related to LDL and lipoprotein clearance, as shown by proteomics analysis. In fact, reduced levels of ApoB100, an important constituent of LDL, have been reported in mice with liver p63 knockdown [14]. This suggests that LDL metabolism could represent another potential underlying mechanism of action of p63 in the liver, although functional experiments will be required to address causality. In particular, because changes in the pathways related to LDL metabolism and apoB100 production in the liver could indicate alterations in how the liver handles cholesterol and lipoproteins. However, the fact that there were no changes in serum cholesterol levels suggests that the changes observed in the liver might be localized or compensated for by other regulatory mechanisms in the body, without significantly affecting the overall cholesterol levels in the blood. These mechanisms could include alternate pathways for cholesterol metabolism, since the body has multiple pathways for cholesterol metabolism, both in the liver and in peripheral tissues. Another possibility might be an efflux of cholesterol from tissues. Cells throughout the body, including those in the liver, can export excess cholesterol to HDL particles for transport back to the liver for excretion. Changes in LDL clearance by the liver may lead to increased efflux of cholesterol from tissues to maintain balance. Of note, we did not detect changes in ki67 and BrDU nor in the development of tumors in the liver of mice with silencing of p63 and fed a CDHFD for 52 weeks. Thus, the degree of inhibition of p63 achieved in this study seems to not induce changes in cell proliferation in the long term, an effect of high importance from a translational perspective. Finally, the major changes in metabolic-related proteins found by proteomics suggest a major metabolic role of TAp63 which could underlie the observed changes in steatosis, inflammation, and fibrosis. Regarding the latter, it is described that TAp63 promotes fibrogenesis of HSCs via metabolic changes in a pathway involving ACC1 [24]. However, we cannot rule out that p63 also exerts direct control of inflammatory and fibrotic pathways. In fact, it is reported that metabolic alterations induced by TAp63 in hepatocytes are mediated by the inflammatory IKKβ signaling pathway [14]. In the future, studies searching for direct transcriptional target genes of p63 and the subsequent activation of signaling cascades will help to better understand how hepatic p63 controls the different components of steatohepatitis.

## 5 Conclusions

In summary, the results presented here implicate p63 and specifically its TA isoform in the progression of steatohepatitis with fibrosis. The inhibition of hepatic TAp63 following either prevention or interventional approaches mitigated steatosis, inflammation, and fibrosis in two different mice models of steatohepatitis with fibrosis, while the overexpression of TAp63 aggravated the liver disease progression. Further proteomics analysis in these mice revealed changes in multiple proteins and pathways, some of which were not previously described to be regulated by p63, indicating that it is acting at different stages of liver disease progression.

## CRediT authorship contribution statement

**Marcos F. Fondevila:** Writing – original draft, Formal analysis, Data curation, Conceptualization. **Eva Novoa:** Formal analysis, Data curation. **Uxia Fernandez:** Methodology, Data curation. **Valentina Dorta:** Methodology, Data curation. **Tamara Parracho:** Methodology, Formal analysis, Data curation. **Henriette Kreimeyer:** Formal analysis, Data curation. **Maria Garcia-Vence:** Methodology, Data curation. **Maria P. Chantada-Vazquez:** Methodology, Formal analysis, Data curation. **Susana B. Bravo:** Methodology, Formal analysis, Data curation. **Begoña Porteiro:** Methodology, Data curation. **Alba Cabaleiro:** Methodology, Data curation. **Mijra Koning:** Data curation, Formal analysis. **Ana Senra:** Methodology, Data curation. **Yara Souto:** Methodology. **Joanne Verheij:** Data curation, Formal analysis. **Diana Guallar:** Writing – review & editing, Methodology. **Miguel Fidalgo:** Writing – review & editing, Methodology. **Abraham S. Meijnikman:** Writing – review & editing, Methodology. **Natalia da Silva Lima:** Methodology, Data curation. **Carlos Dieguez:** Writing – review & editing, Supervision. **Maria J. Gonzalez-Rellan:** Writing – review & editing, Supervision. **Ruben Nogueiras:** Writing – original draft, Supervision, Conceptualization.

## Declaration of competing interest

The authors have nothing to declare.

## Data Availability

Data will be made available on request.
