# Supplementary material for: Inhibition of hepatic p63 ameliorates steatohepatitis with fibrosis in mice
Source: Mol Metab. 2024 May 28;85:101962. doi: 10.1016/j.molmet.2024.101962 (PMC11180345; doi:10.1016/j.molmet.2024.101962)
Supplement: Multimedia component 1 [file mmc1.pdf]

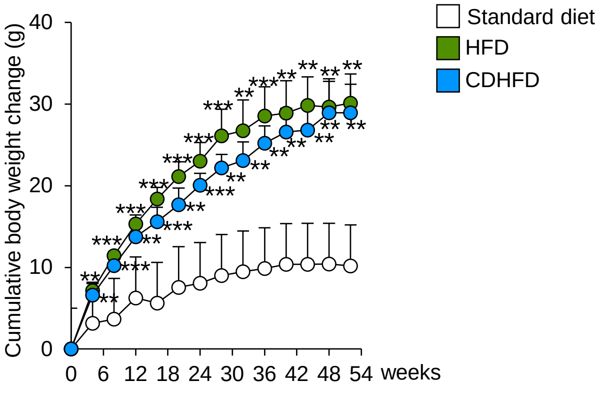

Fig S1. Cumulative body weight change of *wild type* mice fed a standard diet, high fat diet (HFD) or CDHFD for 52 weeks (n=5).

Uncropped blots for Figure 1

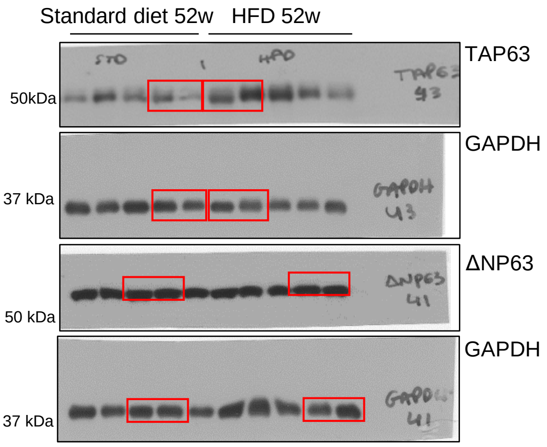

Uncropped blots for Figure 2

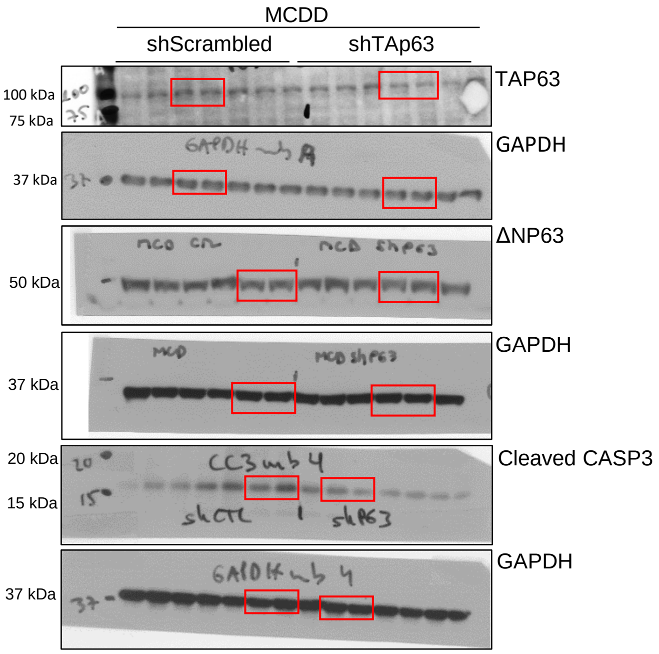

Uncropped blots for Figure 3

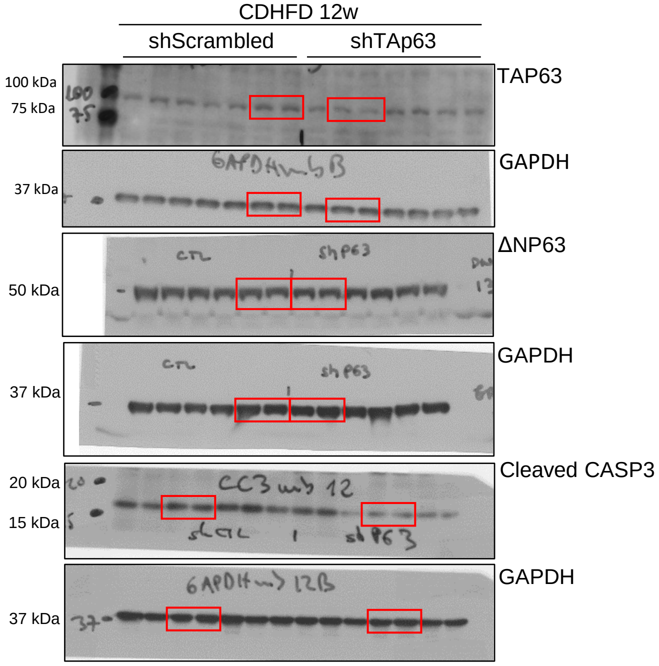

Uncropped blots for Figure 4

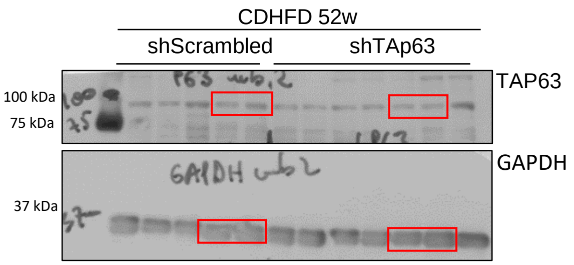

Uncropped blots for Figure 5

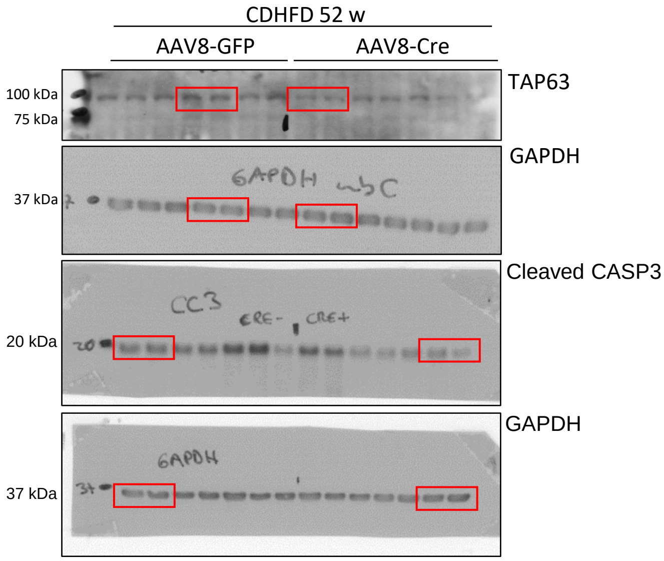

Uncropped blots for Figure 7

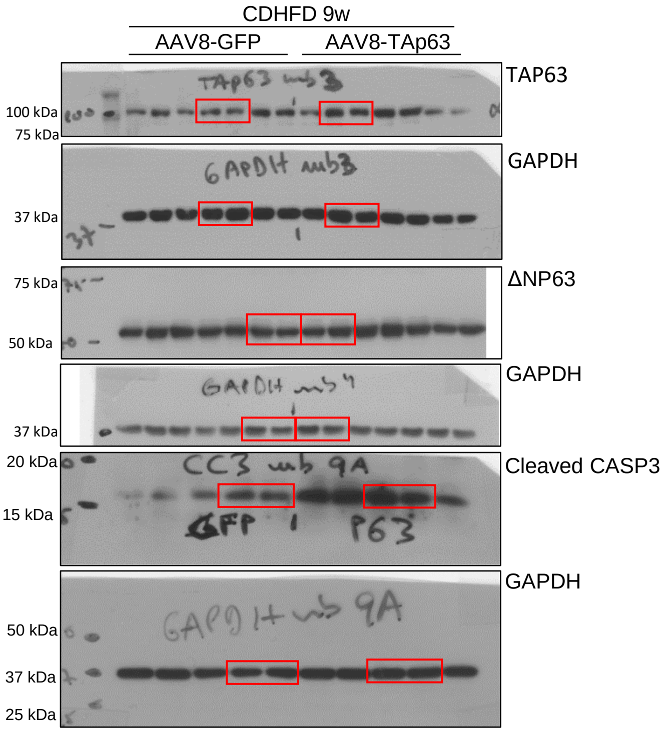

Fig S2. Uncropped blots.

Supplementary Tables

Supplementary table 1. Composition of the rodent diets included in the study.

Diet	**Standard diet**	**High fat diet**	**Choline deficient and high fat diet**	**Methionine- and choline- deficient diet**
Abbreviature	-	HFD	CDHFD	MCDD
Reference	#U8200G10R	#D12451	# D05010402	#A02082002BR
Manufacturer	SAFE Diets	Research Diets	Research Diets	Research Diets
Protein g % (kcal %)	21.4 (15.2)	23.7 (20.0)	23.7 (20.0)	17.0 (16.1)
Carbohydrate g % (kcal %)	56.1(61.3)	41.4 (35.1)	41.5 (35.1)	65.9 (62.7)
Fat g % (kcal %)	5.1 (13.5)	23.6 (44.9)	23.7 (44.9)	9.9 (21.2)
kcal/gm	3.40	4.73	4.74	4.21
**Composition g %**				
Amino acids				
Ala	0.8	0.6	0.6	0.4
Arg	1.4	0.7	0.7	1.2
Asp	1.5	0.3	0.3	0.4
Cys	0.3	0.5	0.5	0.4
Gln	3.4	2.4	2.4	4.0
Gly	1.2	0.4	0.4	2.3
His	0.4	0.5	0.5	0.5
Ile	0.7	0.9	0.9	0.8
Leu	1.3	1.8	1.8	1.1
Lys	1.2	1.5	1.5	1.8
Phe	0.8	0.9	0.9	0.8
Pro	1.2	2.1	2.1	0.4
Ser	0.8	1.2	1.2	0.4
Thr	0.6	0.8	0.8	0.8
Trp	0.3	0.2	0.2	0.2
Tyr	0.6	1.1	1.1	0.5
Val	0.8	1.1	1.1	0.8
Met	+	+	+	-
Choline	+	+	-	-
Nitrogen free extract:				
Starch	33.5	8.5	8.5	14.9
Sucrose	1.8	20.1	20.2	45.1
Fiber	4.0	5.8	5.8	3.0
Fatty acids:				
Palmitic acid	0.8	4.3	4.3	1.1
Stearic acid	0.2	2.3	2.3	0.2
Palmitoleic acid	0.1	0.3	0.3	0.0
Oleic acid	1.0	7.4	7.4	2.5
Linoleic acid	2.5	6.6	6.6	6.0
Alpha-Linolenic acid	0.3	0.5	0.5	0.1

Supplementary table 2. Primers and probes used for gene amplification.

Name	Primer sequence 5´→ 3´
Mus musculus Col1α1	FW cctaatgctgccttttctgc<br>RV atgtcccagcaggatttgag
Mus musculus Col1α2	FW ccgtgcttctcagaacatca<br>RV cttgccccattcatttgtct
Mus musculus F4/80	FW tgcatctagcaatggacagc<br>RV gccttctggatccatttgaa
Mus musculus Hprt	FW aagcttgctggtgaaaagga<br>RV ttgcgctcatcttaggcttt
Mus musculus Il1β	FW actcattgtggctgtggaga<br>RV ttgttcatctcggagcctgt
Mus musculus Il6	FW agttgccttcttgggactga<br>RV tccacgatttcccagagaac
Mus musculus total p63	Mm00495793_m1 (Applied Biosystems)
Mus musculus total TAp63	FW tgccaccctacagtactgcccc<br>RV ctcgcttgtctgggtgctctgc
Mus musculus total ΔNp63	FW gcagccttgaccagtctcactgc<br>RV tccatgctgttcaggagcccca
Mus musculus Tnfα	FW agcccccagtctgtatcctt<br>RV ctccctttgcagaactcagg

Supplementary table 3. Antibodies used for western blot.

Protein target	Manufacturer (catalog number)	Species reactivity	Dilution
TAP63	BioLegend (#938102)	Mouse monoclonal	1:1000
ΔNP63	BioLegend (#619002)	Rabbit polyclonal	1:1000
Cleaved CASP3	Cell Signaling (#9664)	Rabbit monoclonal	1:1000
GAPDH	Merck (#CB1001)	Mouse monoclonal	1:5000

Supplementary table 4. Circulating levels of triglycerides, cholesterol, non-esterified fatty acids (NEFAs), glucose and insulin, and final body weight, liver mass and epidydimal adipose tissue mass in mice fed a standard diet (SD), high fat diet (HFD) or choline deficient and high fat diet (CDHFD) for 6, 12 or 52 weeks (n=5). Data are mean ± SEM. *$p < 0.05$, **$p < 0.01$, ***$p < 0.001$ according to one-way ANOVA followed by Bonferroni post hoc multiple comparison test.

	Diet			Statistical differences		
Variable	SD	HFD	CDHFD	HFD vs SD	CDHFD vs SD	CDHFD vs HFD
	6 weeks					
Triglycerides in serum (mg/dl)	109.46 ± 8.46	171.65 ± 17.74	161.42 ± 11.31	*	*	ns
Cholesterol in serum (md/dl)	119.43 ± 199.07	199.07 ± 17.66	195.08 ± 20.76	*	*	ns
Non-esterified fatty acids in serum (md/dl)	32.47 ± 1.63	42.33 ± 3.15	37.12 ± 2.21	*	ns	ns
Glucose in serum (mg/dl)	127.87 ± 12.41	164.84 ± 10.94	161.17 ± 14.63	ns	ns	ns
Insulin in serum (ng/ml)	0.83 ± 0.32	1.62 ± 0.66	1.53 ± 0.65	ns	ns	ns
Body weight (g)	21.54 ± 0.44	27.71 ± 0.82	27.59 ± 0.89	***	***	ns
Liver mass (g)	1.01 ± 0.04	1.12 ± 0.03	1.16 ± 0.08	ns	ns	ns
Epididymal adipose tissue mass (g)	0.44 ± 0.01	1.06 ± 0.09	1.07 ± 0.25	*	*	ns
	12 weeks					
Triglycerides in serum (mg/dl)	114.87 ± 16.32	242.50 ± 19.42	234.02 ± 18.70	***	**	ns
Cholesterol in serum (md/dl)	125.88 ± 11.61	184.02 ± 8.90	203.21 ± 10.11	**	***	ns
Non-esterified fatty acids in serum (md/dl)	27.48 ± 1.66	44.23 ± 4.65	40.36 ± 4.69	*	ns	ns
Glucose in serum (mg/dl)	124.30 ± 10.28	196.70 ± 11.96	176.70 ± 11.96	**	*	ns
Insulin in serum (ng/ml)	0.85 ± 0.02	2.76 ± 0.76	2.32 ± 0.18	*	*	ns
Body weight (g)	29.40 ± 1.01	38.72 ± 1.33	37.95 ± 1.59	**	**	ns
Liver mass (g)	1.10 ± 0.07	1.52 ± 0.12	1.84 ± 0.12	*	***	ns
Epididymal adipose tissue mass (g)	0.66 ± 0.04	1.50 ± 0.05	1.68 ± 0.12	***	***	ns
	52 weeks					
Triglycerides in serum (mg/dl)	169.41 ± 10.92	327.02 ± 13.13	271.93 ± 12.93	***	***	*
Cholesterol in serum (md/dl)	160.24 ± 12.01	299.60 ± 34.90	281.35 ± 37.00	*	*	ns
Non-esterified fatty acids in serum (md/dl)	47.23 ± 1.77	80.78 ± 5.65	71.32 ± 8.57	**	*	ns
Glucose in serum (mg/dl)	167.35 ± 13.70	298.24 ± 33.31	296.82 ± 11.69	**	**	ns
Insulin in serum (ng/ml)	1.01 ± 0.16	8.50 ± 2.81	8.36 ± 2.15	*	*	ns
Body weight (g)	28.32 ± 1.73	48.51 ± 4.02	48.13 ± 3.78	**	**	ns
Liver mass (g)	1.82 ± 0.17	2.89 ± 0.16	3.65 ± 0.24	**	***	*
Epididymal adipose tissue mass (g)	1.16 ± 0.20	2.68 ± 0.18	2.02 ± 0.09	***	**	*

Supplementary table 5. Circulating levels of triglycerides, cholesterol, non-esterified fatty acids (NEFAs), glucose and insulin, and final body weight, liver mass and epidydimal adipose tissue mass in mice receiving the tail vein injection of lentivirus encoding shRNA TAp63 (n=9) or scrambled (n=9) and fed a CDHFD for 12 weeks. Data are mean ± SEM. *$p < 0.05$, **$p < 0.01$ according to the two-tailed Student's t-test.

Variable	CDHFD 12w Lentivirus-shScrambled	CDHFD 12w Lentivirus-shP63	Statistical differences
Triglycerides in serum (mg/dl)	151.52 ± 6.32	144.25 ± 2.43	ns
Cholesterol in serum (md/dl)	188.19 ± 8.27	195.92 ± 5.45	ns
Non-esterified fatty acids in serum (md/dl)	55.69 ± 1.63	62.97 ± 1.00	**
Glucose in serum (mg/dl)	153.38 ± 10.26	142.71 ± 11.79	ns
Body weight (g)	38.92 ± 1.79	39.60 ± 1.59	ns
Liver mass (g)	1.90 ± 0.14	1.48 ± 0.10	*
Epididymal adipose tissue mass (g)	1.93 ± 0.17	1.99 ± 0.27	ns

Supplementary table 6. Circulating levels of triglycerides, cholesterol, non-esterified fatty acids (NEFAs), glucose and insulin, and final body weight, liver mass and epidydimal adipose tissue mass in mice fed a CDHFD for 40 weeks receiving the tail vein injection of lentivirus encoding shRNA TAp63 (n=5) or scrambled (n=7) and maintained in CDHFD for additional 12 weeks. Data are mean ± SEM. *$p < 0.05$, according to the two-tailed Student's t-test.

Variable	CDHFD 52w Lentivirus-shScrambled	CDHFD 52w Lentivirus-shP63	Statistical differences
Triglycerides in serum (mg/dl)	211.31 ± 16.40	173.65 ± 9.90	ns
Cholesterol in serum (md/dl)	242.27 ± 30.16	276.92 ± 31.05	ns
Non-esterified fatty acids in serum (md/dl)	63.28 ± 5.23	79.56 ± 4.30	*
Glucose in serum (mg/dl)	269.34 ± 27.46	281.59 ± 21.20	ns
Insulin in serum (ng/ml)	2.50 ± 0.27	2.15 ± 0.40	ns
Body weight (g)	52.34 ± 6.01	53.44 ± 2.36	ns
Liver mass (g)	3.09 ± 0.26	2.40 ± 0.15	*
Epididymal adipose tissue mass (g)	2.97 ± 0.32	3.51 ± 0.51	ns

Supplementary table 7. Circulating levels of triglycerides, cholesterol, non-esterified fatty acids (NEFAs), glucose and insulin, and final body weight, liver mass and epidydimal adipose tissue mass in TAp63-floxed mice fed a CDHFD for 40 weeks receiving the tail vein injection of lentivirus encoding adeno-associated virus serotype 8 (AAV8) encoding Cre recombinase (n=9) or GFP (n=9) and maintained in CDHFD for additional 12 weeks. Data are mean ± SEM. *$p < 0.05$, according to the two-tailed Student's t-test.

Variable	CDHFD 52w<br>Flox-TAp63 AAV8-GFP	CDHFD 52w<br>Flox-TAp63 AAV8-Cre	Statistical differences
Triglycerides in serum (mg/dl)	257.37 ± 11.05	279.90 ± 27.78	ns
Cholesterol in serum (md/dl)	200.81 ± 17.20	218.48 ± 24.03	ns
Non-esterified fatty acids in serum (md/dl)	60.50 ± 6.96	83.47 ± 5.37	*
Glucose in serum (mg/dl)	256.24 ± 13.35	205.68 ± 12.15	*
Insulin in serum (ng/ml)	3.08 ± 0.32	4.80 ± 1.29	ns
Body weight (g)	58.76 ± 4.62	56.92 ± 4.24	ns
Liver mass (g)	3.28 ± 0.24	2.52 ± 0.26	*
Epididymal adipose tissue mass (g)	2.49 ± 0.16	1.80 ± 0.24	*

Supplementary table 8. Histological evaluation of liver by NAS in TAp63-floxed mice fed a CDHFD for 40 weeks receiving the tail vein injection of lentivirus encoding adeno-associated virus serotype 8 (AAV8) encoding Cre recombinase (n=5) or GFP (n=5) and maintained in CDHFD for additional 12 weeks. Data are shown as absolute values as percentage.

Histological features	Score	Category definition	CDHFD 52w Flox-TAp63 AAV8-GFP	CDHFD 52w Flox-TAp63 AAV8-Cre
Steatosis grade				
	0	<5%	0 (0%)	1 (20%)
	1	5-33%	0 (0%)	0 (0%)
	2	33-66%	0 (0%)	2 (40%)
	3	>66%	5 (100%)	2 (40%)
Lobular inflammation				
	0	No foci	0 (0%)	0 (0%)
	1	<2 foci per 20X field	2 (40%)	4 (80%)
	2	2-4 foci per 20X field	3 (40%)	1 (20%)
	3	>4 foci per 20X field	0 (0%)	0 (0%)
Hepatocyte ballooning				
	0	None	0 (0%)	2 (40%)
	1	Few balloon cells	5 (100%)	3 (60%)
	2	Many cells/ prominent ballooning	0 (0%)	0 (0%)
Fibrosis stage				
	0	None	0 (0%)	1 (20%)
	1	Perisinusoidal or periportal	2 (40%)	3 (60%)
	2	Perisinusoidal and portal/periportal	3 (60%)	1 (20%)
	3	Bridging fibrosis	0 (0%)	0 (0%)
	4	Cirrhosis	0 (0%)	0 (0%)

Supplementary table 9. Circulating levels of triglycerides, cholesterol, non-esterified fatty acids (NEFAs), glucose and insulin, and final body weight, liver mass and epidydimal adipose tissue mass in mice receiving the tail vein injection of adeno-associated virus serotype 8 (AAV8) encoding TAp63 (n=7) or scrambled (n=7) and fed a CDHFD for 9 weeks. Data are mean ± SEM. *$p < 0.05$, according to the two-tailed Student's t-test.

Variable	CDHFD 9w AAV8-GFP	CDHFD 9w AAV8-TAp63	Statistical differences
Triglycerides in serum (mg/dl)	164.94 ± 18.70	150.45 ± 17.17	ns
Cholesterol in serum (md/dl)	192.14 ± 9.05	189.43 ± 8.86	ns
Non-sterified fatty acids in serum (md/dl)	47.41 ± 6.59	43.70 ± 2.88	ns
Glucose in serum (mg/dl)	126.48 ± 17.04	152.46 ± 20.79	ns
Insulin in serum (ng/ml)	3.90 ± 0.67	3.76 ± 0.87	ns
Body weight (g)	34.87 ± 2.38	35.99 ± 1.99	ns
Liver mass (g)	1.23 ± 0.05	1.50 ± 0.11	*
Epididymal adipose tissue mass (g)	1.53 ± 0.27	1.74 ± 0.23	ns

Supplementary table 10. Histological evaluation of liver staging by NAS in mice receiving the tail vein injection of adeno-associated virus serotype 8 (AAV8) encoding TAp63 (n=7) or scrambled (n=7) and fed a CDHFD for 9 weeks. Data are shown as absolute values as percentage.

Histological features	Score	Category definition	CDHFD 9w AAV8-GFP	CDHFD 9w AAV8-TAp63
Steatosis grade				
	0	<5%	2 (40%)	0 (0%)
	1	5-33%	2 (40%)	3 (60%)
	2	33-66%	1 (20%)	1 (20%)
	3	>66%	0 (0%)	1 (20%)
Lobular inflammation				
	0	No foci	2 (40%)	0 (0%)
	1	<2 foci per 20X field	3 (60%)	5 (100%)
	2	2-4 foci per 20X field	0 (0%)	0 (0%)
	3	>4 foci per 20X field	0 (0%)	0 (0%)
Hepatocyte ballooning				
	0	None	4 (80%)	1 (20%)
	1	Few balloon cells	1 (20%)	4 (80%)
	2	Many cells/ prominent ballooning	0 (0%)	0 (0%)
Fibrosis stage				
	0	None	2 (40%)	0 (0%)
	1	Perisinusoidal or periportal	3 (60%)	5 (100%)
	2	Perisinusoidal and portal/periportal	0 (0%)	0 (0%)
	3	Bridging fibrosis	0 (0%)	0 (0%)
	4	Cirrhosis	0 (0%)	0 (0%)
